# Toward Predictive Understanding of Fatigue Crack Nucleation in Ni-Based Superalloys

**DOI:** 10.1007/s11837-017-2307-9

**Published:** 2017-03-24

**Authors:** Jun Jiang, Fionn P. E. Dunne, T. Ben Britton

**Affiliations:** 1 Department of Mechanical Engineering, Imperial College London, London, SW7 2AZ UK; 2 Department of Materials, Imperial College London, London, SW7 2AZ UK

## Abstract

Predicting when and where materials fail is a holy grail for structural materials engineering. Development of a predictive capability in this domain will optimize the employment of existing materials, as well as rapidly enhance the uptake of new materials, especially in high-risk, high-value applications, such as aeroengines. In this article, we review and outline recent efforts within our research groups that focus on utilizing full-field measurement and calculation of micromechanical deformation in Ni-based superalloys. In paticular, we employ high spatial resolution digital image correlation (HR-DIC) to measure surface strains and a high-angular resolution electron backscatter diffraction technique (HR-EBSD) to measure elastic distortion, and we combine these with crystal plasticity finite element (CPFE) modeling. We target our studies within a system of samples that includes single, oligo, and polycrystals where the boundary conditions, microstructure, and loading configuration are precisely controlled. Coupling of experiment and simulation in this manner enables enhanced understanding of crystal plasticity, as demonstrated with case studies in deformation compatibility; spatial distributions of slip evolution; deformation patterning around microstructural defects; and ultimately development of predictive capability that probes the location of microstructurally sensitive fatigue cracks. We believe that these studies present a careful calibration and validation of our experimental and simulation-based approaches and pave the way toward new understanding of crack formation in engineering alloys.

## Introduction

Materials science and engineering focus on understanding how defects and microstructure control performance. In structural materials, our focus is on strength and failure, and the role of grains is critical. Deformation processes at the grain level are captured within the domain of crystal plasticity, and these processes can be characterized through experiment and simulation. In engineering alloys, the evolution of the microstructure, such as grains, damage, or dislocation structures, is critical in many significant industrial problems, such as fatigue crack nucleation.^1^^–^ 3

Recent progress in understanding the evolution of the microstructure during mechanical deformation has been afforded through the exploitation of direct transmission electron microscope (TEM) dislocation characterization. Experimental results from TEM are qualitatively beautiful and often quantitatively useful, such as the nature and structure of dislocations within a dislocation wall^4^^,^ 5 or the dislocation types, spacings, and reactions involved in a single-dislocation grain–boundary interaction.^6^^,^ 7 These observations are useful snapshots of local behavior, but the volume of material probed is necessarily limited,^1^ and therefore, capturing the grain behavior at longer length scales and from bulk samples is of limited use.

The performance of engineering alloys often involves understanding deformation across multiple length scales and often the interaction of deformation near heterogeneous interfaces such as grain boundaries. This level of detail is essential to predict component performance, and therefore, features at the micrometer length scale are often critical, such as for microstructurally sensitive processes involved in crack nucleation. This motivates extension of insight at the nm length scale, obtained from TEM thin foils extracted from lightly deformed samples, to studies that include understanding of deformation across multiple grains, to higher strains and at larger length scales.

In the past five years, significant progress has been made to improve our understanding of plasticity in polycrystals through the development of new experimental techniques, e.g., cross-correlation based high angular resolution (HR-EBSD)^8^^,^ 9 and high spatial resolution digital image correlation (HR-DIC),^10^^–^ 13 which provide the means to measure elastic and plastic deformation gradients within polycrystals, including elastic strain, lattice rotation, plastic strain, and continuum rotation.

The results obtained can be used directly to validate the developed crystal plasticity finite element (CPFE) modeling results.^14^^–^ 16 The combination of these micromechanical techniques provides a powerful tool to reveal the underlying deformation mechanisms within crystals and enhances our understanding of plasticity in polycrystals. The aim of this review article is to summarize and draw together the micromechanical studies carried out within our research group based on these three key techniques to address the fatigue crack nucleation problem.

## Characterization, Modeling, and Test Methods

HR-EBSD enables the determination of elastic strain and the lattice rotation tensors based on polar decomposition.^17^ It also enables the study of geometrically necessary dislocation (GND) structures, that is, those dislocations that give rise to a net Burger closure failure. Lattice rotation gradient fields are employed to estimate the GND content stored within crystals,^18^^,^ 19 providing improved angular resolution (~100×) over the conventional Hough-based method.^19^ The HR-EBSD can also be used to estimate total dislocation density through statistical analysis of distribution of shear stress within a probed area.^20^

Digital image correlation (DIC) is an optical method to spatially resolve displacements of features across the surface of a sample. The image analysis technique is used to track these displacements with high precision, through analysis of regions of interest (ROIs), ultimately giving strain maps of a high resolution. We have developed a simple and effective surface coating method that was first reported by Jiang et al.^17^ where nanosize (~250 nm) silica particles were stably and uniformly applied onto the free surface of the samples.^17^^,^ 21 ^,^ 22 These nanosize particles enable spatial high-resolution strain measurements as these high-contrast features are imaged at high-image magnifications. SEM-based imaging of the surface displacement field using our in-house MATLAB-based DIC codes enables us to obtain high-resolution, full-field strain maps.

Crystal plasticity finite element methods model true representations of the single-, oligo-, and polycrystal Ni samples. The models have been well reported on in the literature and, hence, are not elaborated on here, but readers may find details in Refs. 14, 15, and 23. The crystal slip rule is as follows:

1 $$ \dot{\gamma }^{i} = \rho_{g}  b^{{i^{2} }} \nu  { \exp }\left( { - \frac{\Delta F}{kT}} \right)\sin \text {h}\left( {\frac{{\left( {\tau^{i} - \tau_{c} } \right)\Delta V^{i} }}{kT}} \right) $$

where the shear strain rate on each slip system, $\dot{\gamma }^{i}$, is linked through a sinh term to slip activity when the shear stress on the slip system, $\tau^{i}$, exceeds the critical resolved shear stress, $\tau_{c}$, subject to an activation volume $\Delta V^{i}$ and thermal contributions with Boltzmann’s constant multiplied by temperature, i.e., $kT$. The thermal activity of crystal slip is encompassed within an exponential, describing a Gibbs-based statistical approach in capturing the average glide velocity, where $\Delta F$ is an activation energy for the obstacle type. Finally, the prefactors $\rho_{g}  b^{{i^{2} }} \nu$ capture the density of glissile dislocations $\rho_{g}$, their Burgers vector $b^{i}$, and the dislocation jump frequency $\nu$. Many of the required properties are now directly measurable through the use of micromechanical tests.^24^

Mechanical testing was performed on single-crystal CMSX4, oligocrystal MAR-002, and powder metallurgy FGH96 and RR1000 Ni-based superalloys under three-point bend testing, which focuses the maximum tensile stress at a known location for HR-DIC and HR-EBSD analyses.

## Case Studies

### Single- and Oligocrystal Behavior

**Fig. 1:**
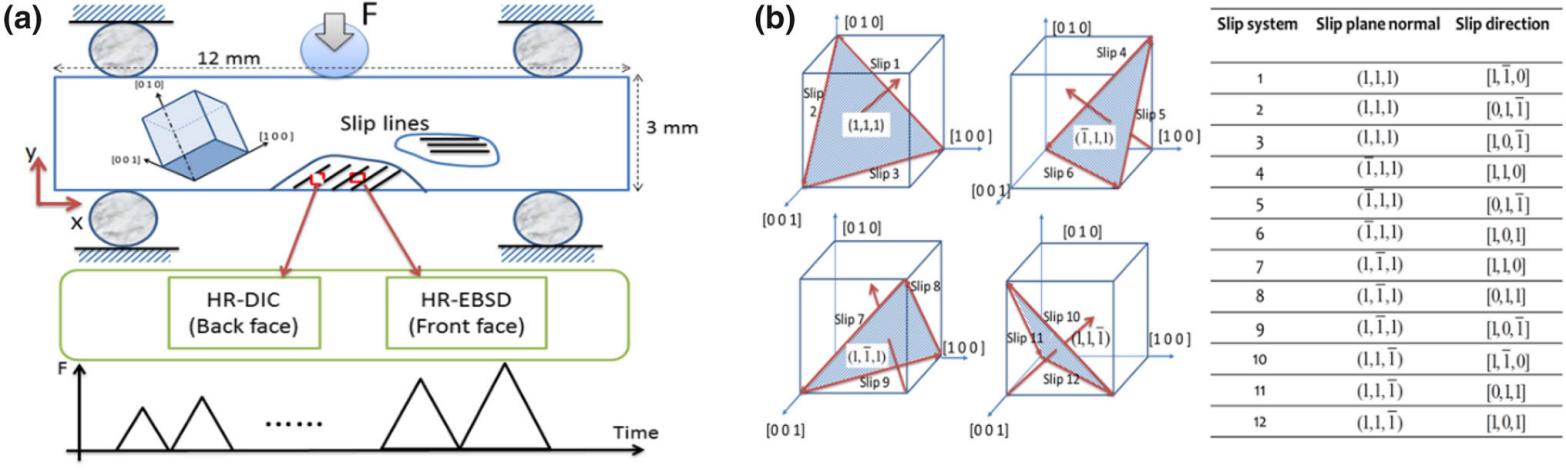
(a) Schematic diagrams showing one single-crystal, three-point beam loading the key regions of interest for slip, the predominant slip activity, the locations and beam faces at which HR-DIC and HR-EBSD characterization have been carried out, and finally, the progressively increasing load cycles applied. (b) Identification of FCC slip directions and normal, and slip direction number designations used throughout this section of the article, shown with respect to an orthogonal RHS (*x*, *y*, *z*) reference system (figure adapted from Ref. 25)

**Fig. 2:**
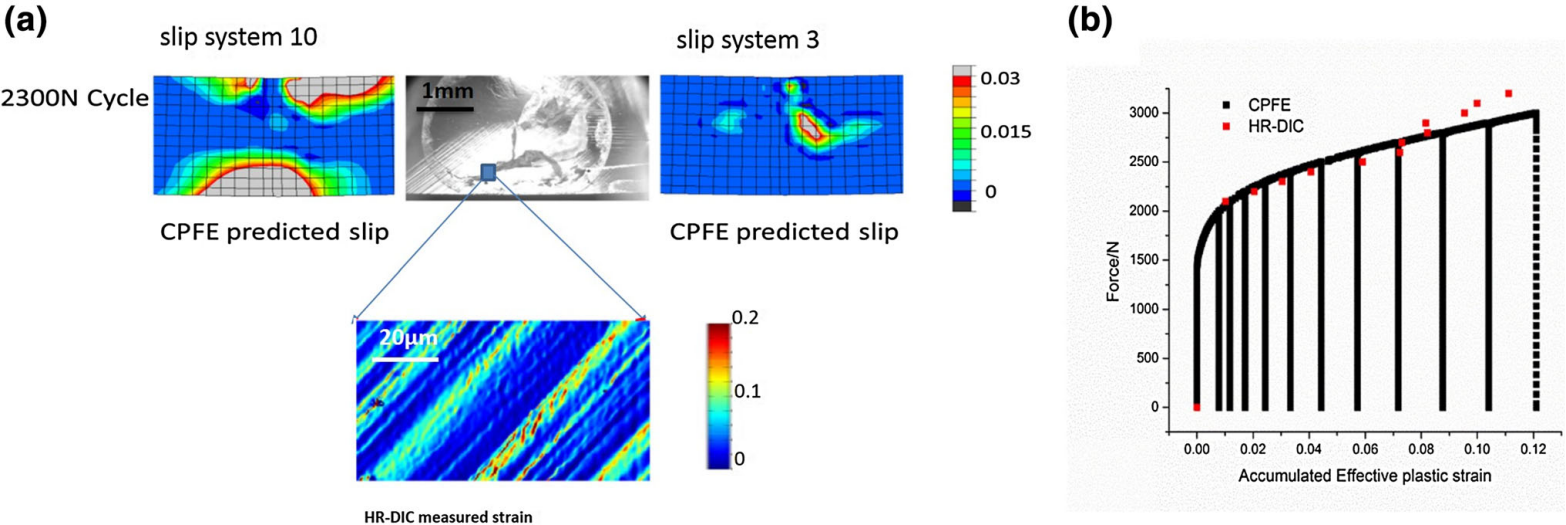
(a) Comparison of the experimentally (HR-DIC) measured and the crystal plasticity predicted slip fields for sample 1 on the DIC face. Slip systems 3 and 10 shown in Fig. 1 are found to predominate, and their spatial distributions are well captured by the crystal plasticity model. (b) The HR-DIC measured and the CPFE determined accumulated plastic strain corresponding to the cycles shown, obtained by averaging the measured and the predicted strains over the region of interest (figure adapted from Ref. 25)

Our first case study addresses the slip in single-crystal samples, which was subjected to an incrementally increasing three-point bending load (as seen in Fig. 1a). The slip activities on the free surface were captured by an optical microscope (OM) as revealed in Figs. 1 and 2, and a microstructurally faithful CPFE model was created to model the plasticity for the given crystal orientation.

From the experiment, two major slip fields are observed: (I) one located within the major tensile region and the extent of this field varies along the length of the beam, as the sample is deformed under three-point bending; and (II) horizontal slip bands operating near the apparent neutral axis on the right of the imaged beam section.

The shape and extent of these fields were well captured by the CPFE modeling, and the results are compared with the OM observed slip activities in Fig. 2a. From the CPFE model, it is apparent that the anisotropic nature of crystal deformation is essential in capturing the nature of plastic slip, even in this simple test, demonstrated by the second slip system activation. The second validation of the operation of these slip systems has been confirmed by using slip trace analysis, and the active slip systems matched well with the CPFE predictions.

The HR-DIC was used to measure the strain field in a region of interest (ROI) of 250 *μ*m × 250 *μ*m size at the middle bottom region of the sample. As revealed in Fig. 2a, captured slip lines roughened and broadened with increasing load. The explicit heterogeneity of dislocation slip (i.e., discrete slip bands) at this length scale is necessarily smoothed out within the CPFE approach, and capturing this would need discrete dislocation modeling. Nevertheless, CPFE can capture the slip fields and the predicted effective strains that agree with those averaged from the HR-DIC measurements (see Fig. 2b). It should be noted that the elliptical ring surface feature in the OM image in Fig. 2a is an artefact as a result of sample preparation. An enlarged OM map of the sample can be found in Ref. 25. The fact that we capture the size, extent, and slip system type for the slip fields in both simulation and experiment, using a single-crystal exemplar, gives confidence about the accuracy of CPFE approach.

**Fig. 3:**
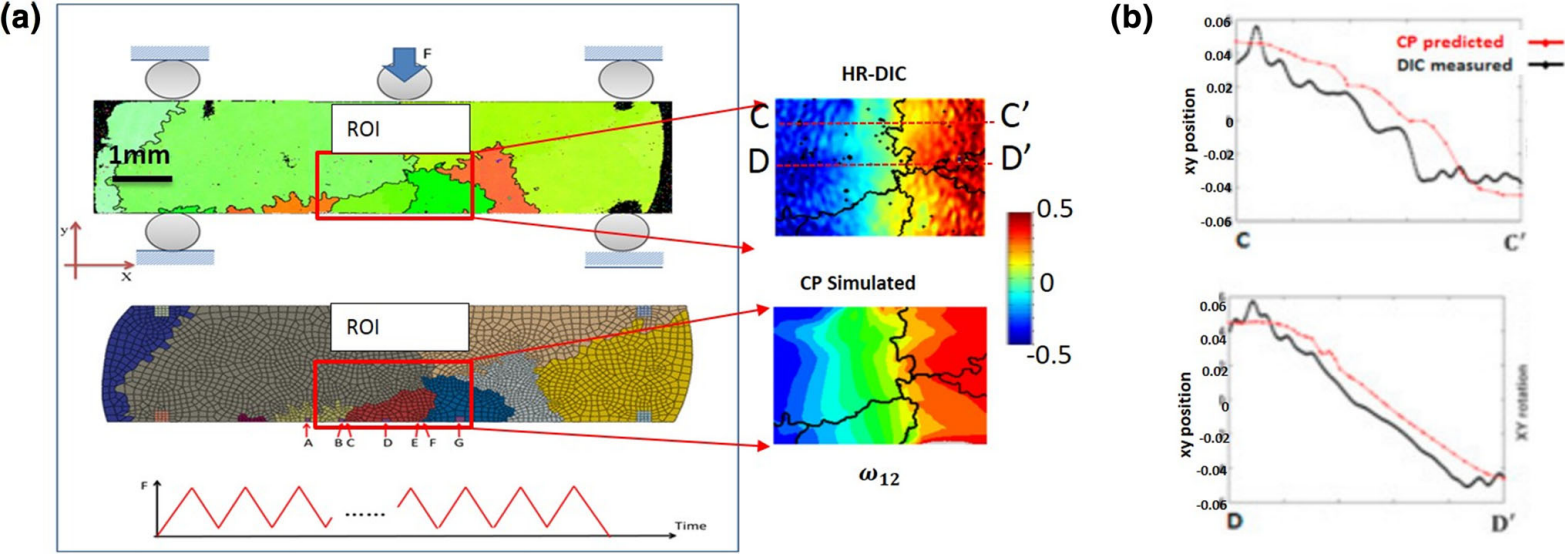
(a) A large-grained Ni oligocrystal sample with a region of interest (ROI) identified and crystallographic orientations of grains 1, 2, and 3 shown, subject to three-point cyclic bend loading shown and the finite element discretization employed for the ROI. The insets show direct comparisons of the HR-DIC and the CPFE in-plane rotation measurement. The associated quantitative comparisons of experiment and simulation of the marked lines CC′ and DD′ are revealed in (b) (figure adapted from Ref. 25)

A more complex challenge is to capture the heterogeneous nature of a crystal slip in a sample with multiple grains. An oligocrystal sample, consisting of six grains within the ROI, is used. The CPFE model is shown in Fig. 3a. Individual in-plane plastic strain and rotation terms measured by HR-DIC at the ROI were compared with the CPFE predicted results. Quantitative and qualitative agreement between experiment and simulation is excellent, as shown by the HR-DIC experiments and CPFE simulations (Fig. 3b).

### HR-EBSD Study of Plasticity in Polycrystals Containing a Second Phase

Nonmetallic inclusions are inevitably mixed into the Ni-based superalloy powders during the powder metallurgy (PM) forming process used to make components, such as turbine disks,^26^^,^ 27 and are often found to be the fatigue crack initiation sites^28^^,^ 29 and limit fatigue life of PM formed components. Inclusion sizes are carefully controlled by filtering out the large inclusions^30^ so that the remaining inclusion sizes are comparable with nickel powder particles, which are approximately the size of several grains.

We have conducted two case studies, one which is focused on following the evolution of the strain state using HR-EBSD, and the second is focused on exploring the surface strain state with HR-DIC. We have cut samples to locate an inclusion within the characterized ROI. Three-point bending tests were carried out with increasing load (HR-DIC sample) and an increasing number of fatigue cycles (HR-EBSD sample).

**Fig. 4:**
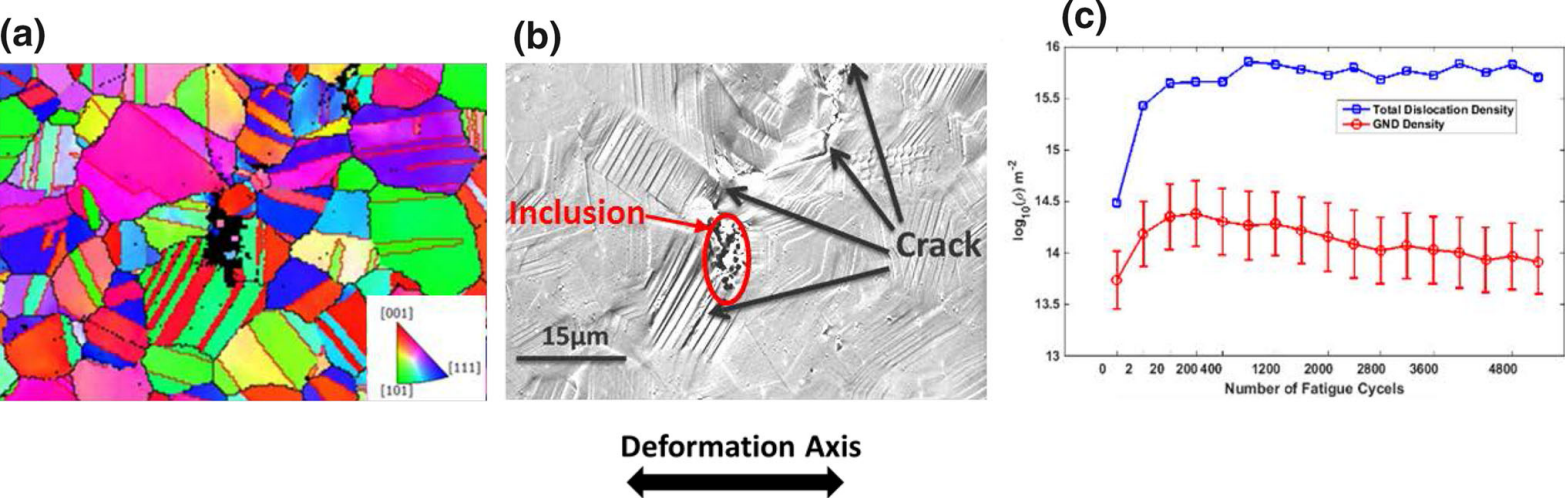
(a) An EBSD crystal orientation map of the ROI with respect to the major deformation axis. (b) A SEM image showing the inclusion and fatigue cracks near it. (c) A statistical analysis of GND and total dislocation density development as a function of the number of fatigue cycles (figure adapted from Ref. 31)

The results from the HR-EBSD study are presented in Fig. 4, which shows characteristic SEM micrographs of this area and that changes in surface topography are microstructurally sensitive as a result of the evolution of the surface slip, shear along the twin boundaries (revealing a stepped morphology with a frequency related to the twin structure), and all these features are observed after only two cycles. A small crack occurred at the inclusion after 20 cycles, which continued to grow through the matrix. Longer cracks (>8 grains) were formed after 5200 cycles.

The evolution of the map-averaged GND and total dislocation density are shown in Fig. 4c. From the total GND density plot, the most striking observation is that the GND density rapidly increases and then shows an apparent decrease. This decrease could be a result of plastic shakedown, where initially high dislocation densities are reduced because of the formation of lower energy structures. Nevertheless, further investigations^31^ on this sample revealed that there was a systematic reduction in GND density in the locally imaged area, as a result of the growth of a carbon film during imaging, which reduces the sensitivity of the GND measurement, and so the reduction of GND density with cycles should be investigated with care. In addition, the HR-EBSD approach to estimating total dislocation density is based on linking the distribution of stresses probed to a distribution of edge dislocations, while the screw type of dislocations is neglected. To address this problem, ECCI is a promising technique to be used as an alternative dislocation characterization method to determine the total dislocation density.^32^^,^ 33

**Fig. 5:**
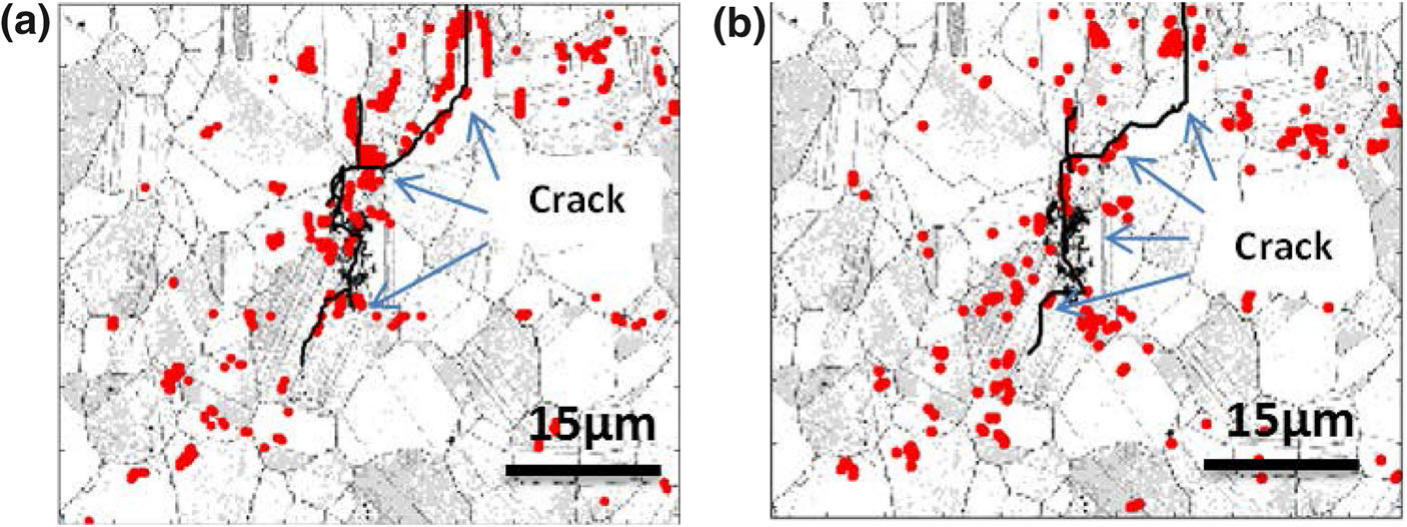
GND density map (a) and grain normalized in-plane shear stress map (b) after two cycles. The dark gray lines represent grain boundaries. Points from the top 5% of GND density distribution, from the entire map, after cycle two are plotted as red dots overlaid on the EBSD image quality map in which the crack path is clearly marked (figure adapted from Ref. 31)

The initial dislocation density was low and homogeneously distributed through the microstructure, and the presence of residual stress gradients was low.^31^ After two cycles, the GND density and residual stress gradient “hot spot” (red) maps are shown in Fig. 5, where points from the top 5% of GND density distribution from the entire map are overlaid on the EBSD image quality (IQ) map. “String” patterns of these hot spots are found to correlate strongly with the formation of the underlying cracks. From this study, it is unclear whether the presence of a high GND density is the precursor to crack formation or whether the high GND density is forming where the grains are being “pulled apart” the most by their local neighborhood.

### HR-DIC and HR-EBSD Study of the Plasticity in Polycrystals with Inclusions

**Fig. 6:**
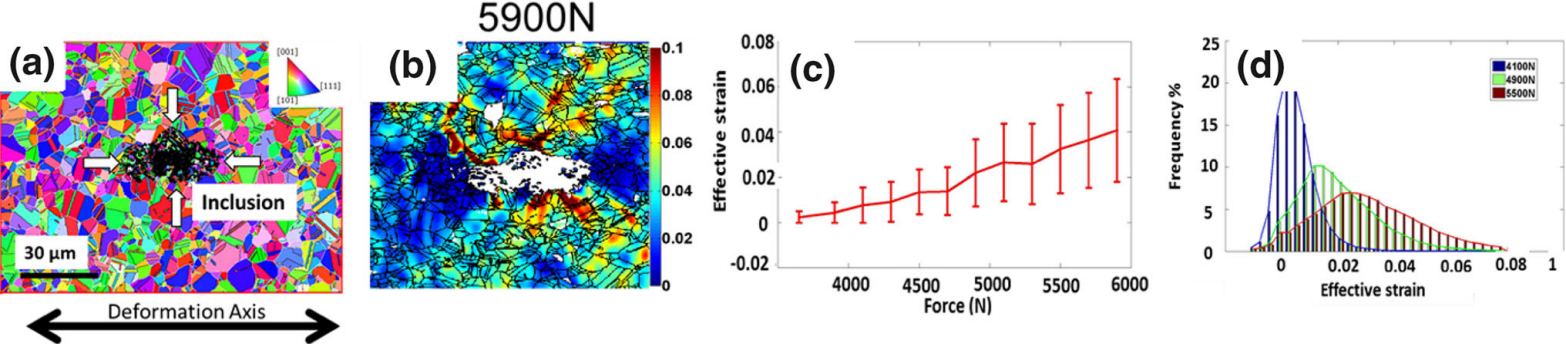
(a) An initial EBSD inverse pole figure map near inclusion with respect to deformation axis. (b) The HR-DIC measured strain distribution near the inclusion at 5900N. (c) The statistical analysis of effective strain distribution and development as a function of load. Three examples of detailed strain distribution are plotted as a histogram and shown in (d) (figure adapted from Ref. 21)

The second sample contains an inclusion in the ROI as shown in Fig. 6. The HR-EBSD analysis was carried out at the beginning and end of the loading process. After each loading cycle, the sample was removed from the rig and placed in a SEM to acquire high-quality micrographs for the HR-DIC analysis. Thus, in this test, we obtained both plastic and elastic distortions at the same region where cracks were formed. Combining with the in situ HR-EBSD analysis mentioned earlier, we were able to link the evolution of elastic and plastic distortions in polycrystals to examine the crack formation process.

The effective strain distribution and evolution are shown in Fig. 6c. Similar to the GND density, the dominant structures within the effective strain maps were formed at the early stage of plasticity. As expected, the map-averaged effective strain values increased with increasing load (this is different to the GND density evolution from the previous example) and the extent of heterogeneity gradually increased as indicated by the error bars in Fig. 6c and d. The effective strain distribution is strongly localized around the inclusion, and it is formed into a “butterfly” shape that is sensitive to both inclusion and the microstructure.

**Fig. 7:**
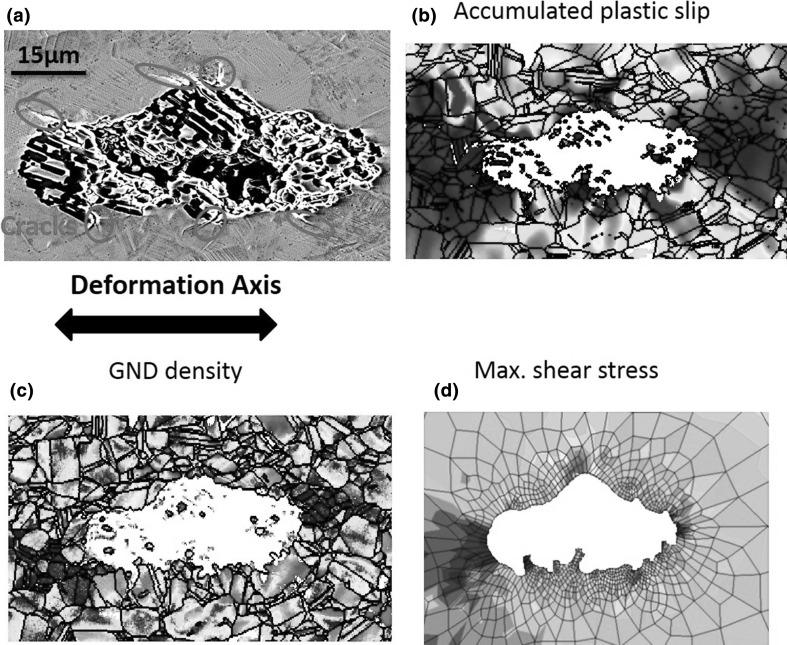
(a) A SEM image shows that six micro-cracks were formed in the nickel matrix at various locations after applied 5900N as highlighted with red circles. (b) The effective strain map overlaid with grain boundary map determined by HR-DIC and EBSD, respectively. (c) The lower bound estimated GND density maps at cracked states (5900N). (d) Finite element analysis of the max shear stress distribution at the inclusion. The nonmetallic inclusion (*E* = 50 GPa, *v* = 0.31) was considered to be softer than the nickel matrix (*E* = 200 GPa, *v* = 0.31) (figure adapted from Ref. 21)

Six micro-cracks within the matrix were observed at locations shown in Fig. 7a. The experimentally measured accumulated plastic slip, GND density, and isotropic elasticity finite element simulation of maximum shear stress at the same area are shown in Fig. 7b, c, and d, respectively. In comparing these three maps, it is interesting to see that the crack sites have high accumulated slip, high maximum shear stress, and high GND density. It is likely that the evolution of these fields and the cracking are related. Yet, detailed inspection of each crack with respect to each field reveals that they are not independent indicators of crack initiation as, for instance, the GND density maps show hot spots toward the interior and away from the inclusion where cracking was not observed. The effective strain map seems to be a more reasonable independent map, and this is supported by a previous study by Dunne et al.^34^ in which they found that the accumulated slip can accurately predict the fatigue crack nucleation sites for most of the studied samples. Nevertheless, in this previous study, not all cracks could be accommodated through a simple evaluation of the accumulated plastic strain (i.e., slip) alone, and so a better match was achieved with a stored energy density criterion for fatigue crack nucleation.

Use of the new criterion allows for consideration of the local stored energy rate (per loading cycle) developed over an area determined by the local dislocation content to define the appropriate length scale with which to define the energy density. It, therefore, places emphasis on the importance of (Griffith-like) stored energy and dislocation density (Stroh’s model). Dunne et al.^34^ posited their model through the following argument:

They considered storage volume $\Delta V$, written in terms of storage area $\Delta A$, statistically stored dislocation (SSD) and GND density, which is given by:

2 $$ \Delta V = \frac{\Delta A}{{\sqrt {\rho_{\text{SSD}} + \rho_{\text{GND}} } }} $$

3 $$ W = \oint {\frac{{\xi \varvec{\sigma} :{\text{d}}\varvec{\varepsilon}^{p} \Delta A}}{{\sqrt {\rho_{\text{SSD}} + \rho_{\text{GND}} } }}} $$

The stored energy per cycle within the volume is then determined to be where $\xi$ is the fraction of the dissipated energy stored in the establishment of dislocation structure, and the integration is carried out over a complete loading cycle. The stored energy rate per cycle is then given by:

4 $$ \dot{G} = \frac{W}{\Delta A} = \oint {\frac{{\xi \varvec{\sigma} :{\text{d}} \varvec{\varepsilon}^{p} }}{{\sqrt {\rho_{\text{SSD}} + \rho_{\text{GND}} } }}} $$

For this study, Eq. 4 must be simplified to enable an indication of the stored energy rate to be estimated from our experimental observations. We do not have a measure of the local absolute stress variation, and so the stress is assumed to be constant and uniform according to the tensile boundary conditions because the local microstructural stress around a given cyclic hysteresis loop is not known. Note that Fig. 7d is a finite element model prediction that does not have explicit local microstructural representation so that the stresses calculated in this work are from assuming Mises plasticity. Although this may not be a good representation at localized regions, e.g., grain boundaries, it is likely to be reasonable for the grain-average level and is therefore a tolerable simplification.

Also, we consider that the SSD density $\rho_{SSD}$ is proportional to the applied plastic strain $\varepsilon$.^14^ We therefore only use the GND density as a stronger influence on the distribution of stored energy density.

This reduces Eq. 4 to give:

5 $$ \dot{G} = \oint {\frac{{I:{\text{d}}\varvec{\varepsilon}^{p} }}{{\sqrt {\rho_{\text{GND}} } }}} $$

The variation of this parameter was found to have a better correlation with the crack locations as compared with either accumulated plastic strain or accumulated GND density independently.

Explicit CPFE modeling of the inclusion and surrounding microstructure is underway, which should provide more insights on the stress distribution near crack nucleation sites and should allow a better examination of this crack nucleation criterion.

### CPFE Model of Polycrystal Containing Second-Phase Particle Fracture and Decohesion

**Fig. 8:**
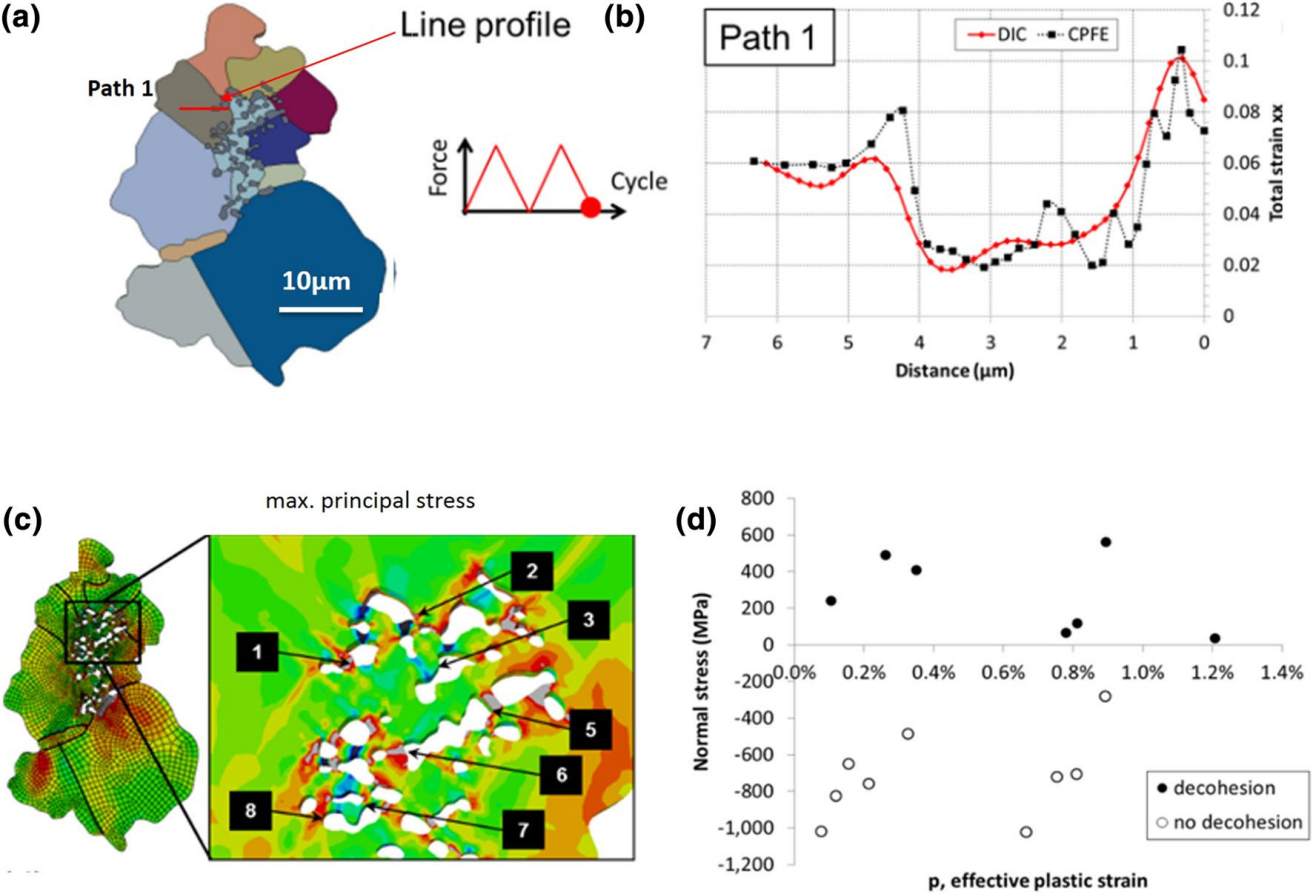
(a) The agglomerate inclusion model and the corresponding paths 1 along which xx strains are obtained by the HR-DIC and from the CPFE simulation extracted for (b) path 1 at the end of the second cycle shown figure inset in (a). The colors in (a) are used to distinguish grains. (c) The CPFE predicted maximum principal stress distribution. (d) A plot of CPFE determined normal stress as a function of effective plastic strain for the observed inclusions (figure adapted from Ref. 35)

The third sample that contained a distribution of nonmetallic inclusions within the Ni matrix was tested and studied by HR-DIC and CPFE.^33^ This inclusion was generated as a result of a different processing route, and so the microstructure and properties of this inclusion structure are different than those discussed, as observed in the explicit microstructure rendered for the CPFE study shown in Fig. 8a.

The comparison of the surface strain fields between each grain surrounding the inclusion captured by HR-DIC and simulated by CPFE, shown in Fig. 8, reveals good agreement between model and experiment and enables the study of the inclusion effects on local plasticity and fatigue crack nucleation.

The experimental observations revealed both decohesion of the matrix and fracture of the particles. As the CPFE model matched the total strain fields from the experiment, the stress fields predicted with the CPFE were used to understand the nature of decohesion and failure of the particles and nearby matrix. The full-field maps representing different reductions of the stress tensor for each point within the modeled region were created, and points near the particles that failed in the experiment were highlighted. The evaluation of the hydrostatic and normal stress fields (and many others that were not reported within the article) revealed that the normal stress perpendicular to the inclusion-matrix interface was found to show the strongest “contrast” and best correlation.

The evaluation of the stress differences between the particles that had failed and those that had not enabled us to predict that the interfacial failure stress for these particles was 1270 MPa (as observed in Ref. 35 in Fig. 14c), which is a result possible only through combined CPFE and HR-DIC measurement.

## Summary and Future Perspectives

The aim of this research effort was to develop predictive capability for fatigue crack nucleation in polycrystalline metallic materials. We designed our series of experiments to build complexity step by step and to validate each microstructural feature and experimental technique in turn and have ultimately achieved a good match between experiment and simulation for crack nucleation near inclusions in polycrystal Ni-based superalloys. The correlative approach that comprises a controlled series of increasingly complex microstructures coupled with HR-EBSD, HR-DIC, and crystal plasticity simulations has enabled calibration and validation of each approach.

A significant body of evidence from direct measurement has now been established for Ni single, oligo-, and polycrystals, as well as for polycrystals containing agglomerates, that no single microstructural quantity, be it local slip accumulation, type III stress, or density of GNDs, in its own right provides a persuasive link to fatigue crack nucleation. Although the development of local slip has been found to be a prerequisite for fatigue crack nucleation, in keeping with much earlier work for the need for persistent slip band (PSB) formation, it is argued to be a necessary rather than a sufficient process. We have found many instances, however, within the experimental programs reported, where highly localized slip does not give rise to crack nucleation. Nonetheless, the contemporaneous utilization of HR-EBSD, HR-DIC, and CPFE modeling has made clear that the highly anisotropic activation of slip, its inhomogeneous development, and its distribution can be effectively captured with CP modeling. Hence, highly localized slip accumulations, progressively increasing over cyclic loading, local type III stresses, and GND densities (within the constraints outlined earlier) are beginning to become quantitatively predictable such that refocus on quantitative prediction of fatigue crack nucleation is appropriate. A new quantity that seems to have merit is found to be a local stored energy density discussed in this article. The evidence presented is limited to the Ni agglomerate polycrystal where the multiple locations of crack nucleation can be captured with the stored energy approach. Nevertheless, the results of other continuing work have revealed that the locations of crack nucleation can be correctly captured in single and oligocrystals and that the experimentally observed scatter in cycles to fatigue crack nucleation can also be captured with this approach in large-grained Ni RS5 alloy.^36^

Equally important as fatigue crack nucleation, but as of yet elusive, is the establishment of quantitative mechanistic understanding of the processes by which microstructurally sensitive fatigue cracks, once nucleated, subsequently grow. The paths they take, be they in transgranular or intergranular (and often both) modes, the rates at which growth occurs in these modes, taking due account of the highly anisotropic nature of transgranular growth in, for example, HCP crystals, all remain to be understood. The role of interfaces, grain or twin boundaries, or phase interfaces all influence crack growth rates and remain to be explained. These are some of the many challenges that remain and provide interesting opportunities for future research.

As shown in this study, the understanding of plasticity in polycrystals at room temperature has been significantly improved through the HR-EBSD, HR-DIC, and CPFE techniques. In-service operating conditions of high-value materials such as aero-engine materials are often at elevated temperatures, which involve viscoplasticity where creep will also be an important deformation mode to be considered. Therefore, the characterization and modeling tools need to be further developed to measure and predict crystal behaviors at high temperatures.

## Footnotes

1 Assuming that it is possible to study a 100-nm-thick 10 × 10-*µ*m^2^ foils in one half day of TEM time, it would take a billion years of TEM time to study the volume of material contained within a little finger.
